# Driving Factors for the Evolution of Species-Specific Echolocation Call Design in New World Free-Tailed Bats (Molossidae)

**DOI:** 10.1371/journal.pone.0085279

**Published:** 2014-01-14

**Authors:** Kirsten Jung, Jesús Molinari, Elisabeth K. V. Kalko

**Affiliations:** 1 Institute of Experimental Ecology, University of Ulm, Ulm, Germany; 2 Departamento de Biología, Facultad de Ciencias, Universidad de Los Andes, Mérida, Venezuela; 3 Smithsonian Tropical Research Institute, Balboa, Panama; University of Southern Denmark, Denmark

## Abstract

Phylogeny, ecology, and sensorial constraints are thought to be the most important factors influencing echolocation call design in bats. The Molossidae is a diverse bat family with a majority of species restricted to tropical and subtropical regions. Most molossids are specialized to forage for insects in open space, and thus share similar navigational challenges. We use an unprecedented dataset on the echolocation calls of 8 genera and 18 species of New World molossids to explore how habitat, phylogenetic relatedness, body mass, and prey perception contribute to echolocation call design. Our results confirm that, with the exception of the genus *Molossops*, echolocation calls of these bats show a typical design for open space foraging. Two lines of evidence point to echolocation call structure of molossids reflecting phylogenetic relatedness. First, such structure is significantly more similar within than among genera. Second, except for allometric scaling, such structure is nearly the same in congeneric species. Despite contrasting body masses, 12 of 18 species call within a relatively narrow frequency range of 20 to 35 kHz, a finding that we explain by using a modeling approach whose results suggest this frequency range to be an adaptation optimizing prey perception in open space. To conclude, we argue that the high variability in echolocation call design of molossids is an advanced evolutionary trait allowing the flexible adjustment of echolocation systems to various sensorial challenges, while conserving sender identity for social communication. Unraveling evolutionary drivers for echolocation call design in bats has so far been hampered by the lack of adequate model organisms sharing a phylogenetic origin and facing similar sensorial challenges. We thus believe that knowledge of the echolocation call diversity of New World molossid bats may prove to be landmark to understand the evolution and functionality of species-specific signal design in bats.

## Introduction

Echolocation calls primarily evolved for spatial orientation and in many cases for the detection, classification, and localization of prey e.g. [1], [2]. Sensory constraints imposed by the foraging habitat (uncluttered, background-cluttered, or highly cluttered space), foraging strategy (aerial-hawking, perch hunting, or gleaning), and prey type (stationary, or moving) are thus considered among the major selection pressures affecting echolocation call design in bats [3]–[6]. Bats facing similar ecological and sensorial challenges often share similar adaptations in their echolocation systems [7].

Besides ecological and sensorial constraints, phylogeny plays an important role in shaping echolocation call design. This is evident at the family level [8], [9], and is also proposed to take place at the genus level, as shown for the Emballonuridae, in which congeneric species possess a very similar echolocation call structure [10], [11]. Echolocation call frequency is further affected by allometric scaling, with larger bat species typically calling at lower frequencies than smaller bat species [12]–[15]. Just recently, it has also been suggested that differences in the frequency of echolocation calls are driven by the necessity of bats to adapt their signals to obtain an optimal acoustic field of view [16].

There is also accumulating evidence that, in addition to orientation and prey acquisition, echolocation signals have a communicative function. Besides the recognition of individuals [17], [18] and their sexes [19], eavesdroppers likely use echolocation signals to distinguish conspecific from heterospecific individuals [20], thus simplifying the finding of good hunting grounds [21], [22], roosting sites [23], and mates [24]. However, it is still highly debated whether a communicative potential favors a differentiation of species-specific echolocation call design among coexisting bat species [1], [25], [26].

At least 9 genera and 37 species of free-tailed bats (Molossidae) occur in the New World, from Canada (SW British Columbia) and the United States (NW, central, and SE states), to southern Chile and Argentina, including the Antilles [27]–[29]. Molossids are a diverse family of bats with numerous species occurring in tropical and subtropical regions, and with much fewer species occurring in temperate regions [28]. They typically possess long and narrow wings (high aspect ratio and wing loading), which imply high flight speeds and rather low maneuverability [30]. With the exception of *Molossops temminckii* [31], New World molossids are known to forage in open space above forest canopies, or over open landscapes [32], [33], thus facing the similar sensorial challenge to navigate far away from obstacles and to detect sparsely distributed aerial insect prey in wide open space [4], [34]. Many molossids possess a high plasticity in search call design, which involves frequency shifts [35], [36], alternation of peak frequencies e.g. [32], and alternation of upward and downward modulated calls within call sequences [31], [37].

Here, we present an unprecedented dataset of search phase echolocation calls of 18 potentially co-existing species of New World molossids, representing 8 genera, and evaluate possible evolutionary factors explaining species-specific differences in echolocation call design within these bats. To limit the effects of intra-specific variability in our analyses, we focus our attention only on calls produced during the search phase [4]. As most molossids face similar sensorial challenges for navigation, we expected echolocation calls to reveal a high similarity, and a typical design of open-space foragers, i.e., low frequencies, shallow-modulation, long call duration, and long pulse intervals [4], [7], [38]. We further expected that differences within this open-space echolocation design could be explained by phylogeny and allometric scaling. Thereby, we hypothesized that, if phylogeny plays a role in shaping echolocation within the family level, closely related species should have similar echolocation call structure, whereas distantly related species should diverge in structural signal features. In addition we expected that, due to allometric scaling, echolocation parameters (namely frequency, call duration, and pulse interval) of molossids vary with body size. We further hypothesized that frequency differences among molossid species potentially allow resource partitioning, and we evaluated this hypothesis by means of a prey perception model. Finally, we consider the possibility that social communication contributes to differences in echolocation call design among molossid species, and argue that intraspecific plasticity might be key to allow social communication within and among species.

## Materials and Methods

### Ethical statement

Recordings of molossid echolocation calls were obtained in Costa Rica, Panama, Venezuela, Bolivia, and Brazil over a period of 10 years. For acoustic species identification in Costa Rica (Permit number: 15361) and Panama (Permit number: SE/A-59–03), we captured individuals that we identified to species, weighed and measured (body mass, forearm length), and subsequently released while recording their vocalizations. Great care was taken that individuals were active to assure independent departure and good flight ability. Reference recordings were conducted in large clearings (20–30 m width) and released animals were directed towards open space. Circling animals above the clearing allowed us to record typical search calls. In addition, only in the initial phase of the study and exclusively in Venezuela, in full compliance with Venezuelan and international laws and ethic codes, we obtained some crucial reference recordings of high-flying individuals (up to 60–80 m above ground) that were collected with a shot-gun and custom-made ammunition containing very small lead pellets. This was done with all the necessary permits in hand when field work was carried out, issued by the Ministerio del Ambiente y de los Recursos Naturales Renovables (Permit number: 15-00811, explicitly including the permission for collecting bat museum specimens using firearms; 15-00855 acoustic recordings). The specimens involved either died instantaneously, or were euthanized quickly and humanely by means of cervical dislocation followed by thoracic compression, as recommended for small mammals by the Guidelines of the American Society of Mammalogists for the use of wild mammals in research (ASM), the American Veterinary Medical Association Guidelines on Euthanasia (AVMA), and the Recommendations for Euthanasia of Experimental Animals, Part 2, European Community Council Directive 86/609/EEC (EEC). Shooting is considered acceptable when other collecting methods are not possible, provided that the firearm and ammunition are appropriate for the species of interest so that the animals are killed swiftly (ASM, EEC). Cervical dislocation and thoracic compression result in a more rapid and painless loss of consciousness that other forms of euthanasia, and are carried out without previous steps of sedation or anesthesia to avoid additional distress or pain to the animal (ASM, AVMA, ECC). Voucher bat specimens so obtained are being kept in the Universidad de Los Andes.

Finally we obtained recordings of free flying molossids in Bolivia, taken within the framework of the Project “Murciélagos de la Sabana del Beni” (Permit number ICA-CBG-UMSS-638/05), in Brazil within the framework of the project Biological Dynamics of Forest Fragments Project (BDFFP Project number 065/98), and in Panama, where we systematically obtained recordings of high flying molossid bats in open space (Permit number: SE/A-59-03). Passive acoustic monitoring is a non-invasive method but crucial to obtain an adequate insight about the natural variability of echolocation calls.

### Acoustic equipment and data analysis

**Figure 1:**
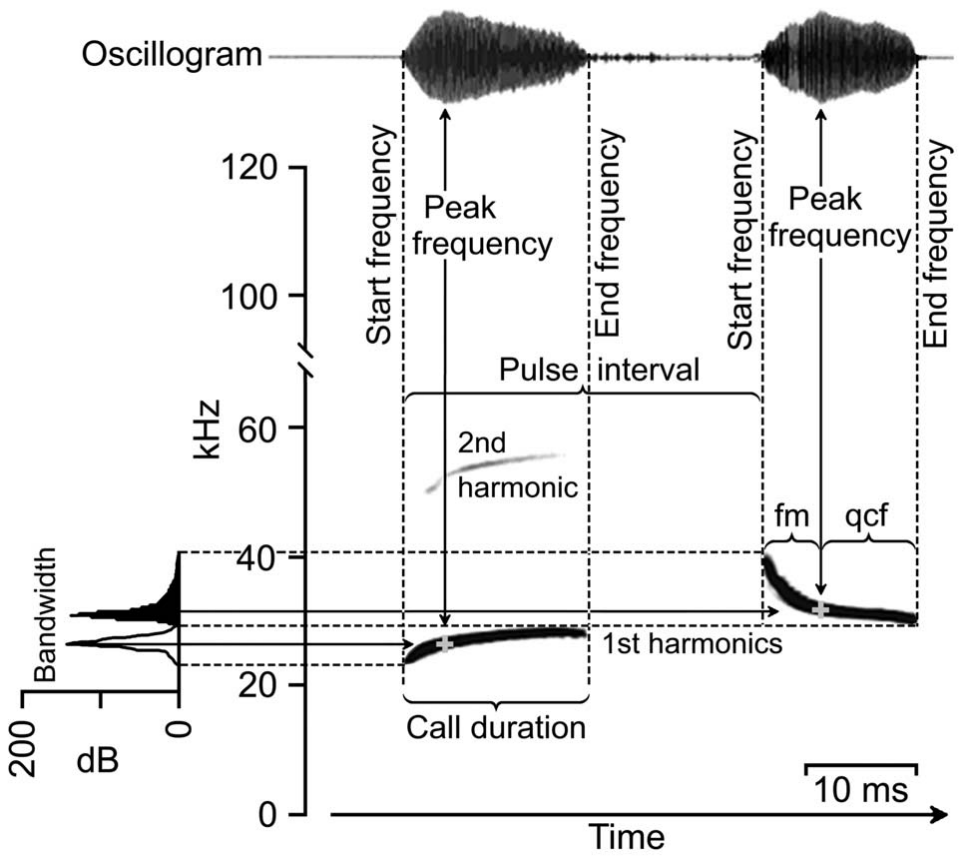
Measurement of echolocation call parameters. Spectrogram of *Promops centralis* (oscillogram, spectrogram, and power spectrum), with upward and downward modulated echolocation calls in search phase, illustrating measurement points of echolocation call parameters used in the analyses.

We gathered reference recordings using several different custom-made time expansion (Delayline: sample rate 312 kHz/12 bit, Animal Physiology, University of Tuebingen. Laar Bridge Box: sample rate 400 kHz/8 bit, AKG Acoustics, Heilbronn, Germany) and real time (PC-Tape: sample rate 500 kHz/16 bit Animal Physiology, University of Tuebingen. Ultra sound gate: sample rate 500 kHz/16 bit, Raimund Specht, Avisoft Bioacoustics, Berlin) acoustic recording devices. We analyzed sound by means of Avisoft Saslab Pro software (Versions 3.95 and 4.34, Raimund Specht, Berlin) using a Hamming window and a frequency resolution of 800–940 Hz, and a time resolution of 0.06–0.08 ms (FFT  = 512 or 1024, overlap 93% or 98% respectively). As we focused our analysis on interspecific differences in search flight echolocation call design, we excluded from the analysis faint calls (signal to noise ratio <10 dB), as well as approach and terminal phase calls. We measured echolocation call parameters such as start, peak, and terminal frequency in the spectrogram window (Figure 1), and calculated call duration, pulse interval, bandwidth including modulation (upward  =  positive values; downward  =  negative values), duty cycle (call duration/pulse interval *100 [%]), and repetition rate (1000 ms/pulse interval [Hz]). In addition, we discriminated between steeper frequency-modulated (fm) components, and narrowband, quasi-constant frequency (qcf) components, using peak frequency of the signals as an inflection point between components. We then calculated the proportional duration (ms) and sweep rate (Hz/ms) of quasi-constant (qcf) versus frequency modulated components in the signal as a measure of call structure. Based on differences in peak frequency (>2 kHz) and structure, we assigned echolocation calls within recording sequences to call types. For our measurements, we considered only the first harmonic, as in molossids it contains most of the sound energy.

To reveal possible differences in the echolocation call design among species and genera, we conducted a permutational multivariate analysis of variance based on Euclidean distances (Adonis, vegan package). Species and genera were included as fixed factors, and call types as random factors. Parameter contribution to the multivariate distances of species and genera were assessed by additional sequential tests. Using the function betadisper (vegan package), we further investigated within-group (species and genera) dispersion of variances in echolocation call parameters. Both analyses were based on mean values per call type within recordings to include echolocation variability of species, while correcting for possible pseudo-replication. Significance was assessed based on 1000 permutations.

We further investigated if structural parameters alone reflect phylogenetic relatedness, and performed a Principal Coordinate Analysis (PCoA) of Euclidean distances. Hereby, we assumed members of the same genera to be monophyletic, which is in accordance with the most recently published molecular phylogeny of molossids [39]. We excluded frequency information and sequential time information (SF, EF, PI, RR; Table 1, 2 & 3) from this analysis, and thus included only parameters representing call structure (BW, CD, D, SR, S; Table 1, 2 & 3). We limited this analysis to the call type with lowest frequency to be most conservative in the representation of the species-specific call structure. Assessment of the significance of the differences among genera were based on the dispersion of variances using 1000 randomizations (betadisper; vegan package).

**Table 1:** Search flight call parameters for the genera *Molossops*, *Neoplatymops*, and *Molossus*.

Call type/structure	SF/EF/BW (kHz)	CD/PI/D (ms)	SR/S (Hz/ms)	DC (%)	RR (Hz)	NC/NS
*Molossops temminckii*						
Low/fm up qcf	42.8±2.6/54.2±1.2/11.4±1.7	8.3±1.2/79.3±5.4/0.9±0.5	1417.8±297.5/4.4±1.3	10.8±1.5	12.9±0.9	77/11
High/fm up qcf	45.5±2.4/54.8±1.4/9.3±1.5	7.6±1.1/82.9±3.8/1.2±0.7	1250.0±235.6/4.1±1.9	9.2±1.5	12.2±0.8	74/11
High II/fm down qcf	75.9±9.9/55.4±1.5/20.5±10.3	7.2±1.0/55.2±3.9/1.3±0.7	2740.0±1095.0/16.9±7.5	13.5±2.3	18.6±1.5	18/11
*Molossops neglectus*						
Low/fm up qcf	32.5±3.3/44.3±1.9/11.8±1.9	10.5±0.9/107.2±3.8/1.2±0.7	1132.4±174.6/5.6±2.8	9.9±0.7	9.5±1.6	28/3
High/fm up qcf	38.3±2.4/46.9±0.8/8.5±3.0	9.4±0.4/107.0±17.1/1.3±0.1	911.9±339.4/6.2±6.9	9.1±1.2	9.8±1.5	29/3
High II/fm down qcf	56.2±2.9/48.9±0.2/7.3±3.0	6.1±1.3/62.2±6.5/1.9±0.1	1286.5±669.7/3.3±3.1	9.8±2.0	16.2±1.6	3/3
*Neoplatymops mattogrossensis*						
Low/(fm) qcf down	32.6±1.7/28.2±1.3/4.3±0.7	12.2±2.9/160.9±40.5/0.4±0.1	377.6±119.9/0.3±0.3	8.6±0.7	6.9±1.3	24/3
High/(fm) qcf down	36.9±0.9/33.6±1.2/3.4±1.2	11.9±3.6/105.1±22.5/0.7±0.2	319.0±192.1/1.9±1.1	11.9±4.5	9.8±2.0	15/3
*Molossus molossus*						
Low/qcf down	35.6±0.9/33.5±1.2/2.2±0.8	10.4±1.4/143.1±25.4/0.5±0.1	211.6±85.2/0.3±0.7	8.0±1.4	7.9±1.5	136/17
Middle/qcf down	39.1±0.9/36.8±1.0/2.2±0.6	10.2±1.3/109.2±44.7/0.5±1.9	220.5±52.9/0.4±0.6	10.9±3.3	10.6±2.9	94/17
High/qcf down	42.8±0.8/39.8±1.2/3.0±1.2	10.4±2.2/82.8±12.2/0.5±0.2	285.8±91.3/0.2±0.8	12.9±3.4	12.5±1.6	56/17
*Molossus sinaloae*						
Low/qcf down	31.2±0.7/31.0±0.9/0.2±0.4	7.6±1.3/178.0±50.5/0.8±0.2	26.1±46.1/1.7±1.3	4.8±1.2	6.4±2.1	42/6
Middle/qcf down	34.0±0.6/33.9±0.9/0.2±0.4	7.6±1.5/110.2±13.0/0.6±0.1	26.1±69.6/2.6±1.5	7.2±1.9	9.4±0.9	25/6
High/qcf down	37.4±0.5/36.8±0.8/0.6±0.3	6.6±1.0/88.9±14.4/0.9±0.6	92.5±36.7/0.6±0.5	7.6±0.8	11.7±2.5	11/6
*Molossus currentium*						
Low/qcf down	29.7±1.3/24.4±2.0/4.3±3.7	13.9±1.7/205.8±57.9/0.5±0.1	353.9±205.5/0.4±0.5	7.5±2.1	5.5±1.5	93/14
Middle/qcf down	32.9±1.6/28.2±2.7/4.4±3.0	14.1±1.8/134.9±31.5/0.6±0.2	347.3±222.9/0.9±0.8	12.3±2.9	8.7±1.8	42/14
High/qcf down	35.1±0.7/30.3±2.0/3.2±4.3	14.4±1.9/126.4±34.3/0.9±0.2	271.8±190.3/1.2±0.7	12.8±3.6	8.8±1.9	37/14
*Molossus rufus*						
Low/qcf down	26.3±0.4/24.7±0.6/1.6±0.6	12.7±0.7/409.0±116.3/0.5±0.1	123.7±49.2/1.1±3.7	3.5±1.0	2.8±0.8	41/8
High/qcf down	27.8±0.4/25.7±0.5/2.0±0.5	12.9±1.2/343.7±131.0/0.7±0.2	158.4±39.4/4.2±0.9	4.4±1.3	3.4±1.2	21/8

For abbreviations and statistical results, see footnote in Table 3.

**Table 2:** Search flight call parameters for the genera *Cynomops*, *Promops*, *Nyctinomops*, and *Tadarida*.

Call type/structure	SF/EF/BW (kHz)	CD/PI/D (ms)	SR/S (Hz/ms)	DC (%)	RR (Hz)	NC/NS
*Cynomops planirostris*						
Low/qcf down	28.8±1.3/21.1±2.4/7.6±1.9	16.1±1.9/236.4±79.1/1.0±0.4	473.7±106.4/1.2±0.9	8.4±2.6	5.2±1.6	46/9
High/qcf down	32.9±1.1/24.3±4.6/8.7±4.5	15.9±2.3/165.1±50.8/1.3±0.6	528.1±287.7/0.4±3.0	11.7±4.6	7.2±2.5	32/9
*Cynomops greenhalli*						
Low/qcf down	25.2±1.3/17.4±3.6/7.8±3.1	15.9±2.1/297.2±115.0/1.0±0.6	503.1±209.3/1.2±1.3	7.0±3.1	4.4±1.8	71/14
High/qcf down	29.0±1.5/21.1±4.6/7.9±4.1	14.8±2.4/190.3±61.0/1.3±0.3	529.8±242.1/1.4±1.3	9.1±3.8	6.1±1.9	40/14
*Promops nasutus*						
Low/fm up qcf	32.7±1.3/34.7±1.3/2.0±0.5	11.6±0.6/209.5±21.9/1.4±0.2	167.3±32.0/0.2±2.1	6.0±0.1	5.1±0.5	30/3
High II/fm down qcf	47.0±0.0/37.8±0.0/9.2±0.0	8.3±0.0/105.7±0.0/0.7±0.0	1142.0±0.0/5.0±0.0	7.8±0.0	9.5±0.0	2/3
*Promops centralis*						
Low/fm up qcf	25.8±0.8/28.0±0.7/2.2±0.7	17.8±3.3/276.9±91.2/0.9±0.2	129.9±54.6/2.5±2.6	7.1±2.0	4.1±1.3	12/13
High II/fm down qcf	35.7±6.5/30.4±1.1/8.1±0.7	17.1±7.8/158.9±88.8/0.6±0.1	438.2±364.5/12.3±5.4	11.8±0.6	7.7±2.7	7/9
*Nyctinomops macrotis*						
Low/(fm) qcf down	28.8±4.0/16.7±1.0/12.0±5.1	13.3±1.9/284.7±4.1/1.6±0.1	948.3±512.2/2.6±0.6	5.1±1.0	3.8±0.2	11/2
*Nyctinomops laticaudatus*						
Low/(fm) qcf down	26.7±1.3/23.6±0.9/2.4±0.9	12.5±1.4/393.7±116.7/0.8±0.4	199.9±71.3/3.0±1.4	3.6±1.7	2.8±1.1	63/10
Middle/(fm) qcf down	28.7±1.1/24.2±0.9/4.6±1.6	12.3±1.2/292.9±82.3/0.9±0.3	364.7±101.5/4.9±2.9	4.6±1.5	3.7±0.9	36/10
High/(fm) qcf down]	32.4±1.3/24.9±1.2/7.5±2.0	12.7±2.9/213.6±59.6/1.1±0.2	598.2±191.9/9.2±8.5	6.6±2.6	5.3±2.5	9/10
*Tadarida brasiliensis*						
Low/(fm) qcf down	27.6±3.0/24.4±1.3/3.2±2.5	13.7±1.5/273.1±55.5/0.8±0.3	0.25±0.2/2.5±1.0	0.1±0	3.7±0.7	48/7

For abbreviations and statistical results, see footnote in Table 3.

**Table 3:** Search flight call parameters for the genus *Eumops.*

Call type/structure	SF/EF/BW (kHz)	CD/PI/D (ms)	SR/S (Hz/ms)	DC (%)	RR (Hz)	NC/NS
*Eumops nanus*						
Low/fm qcf down	27.9±0.1/25.2±0.2/2.8±0.3	15.6±2.4/294.6±32.1/0.8±0.3	166.9±40.3/2.0±0.7	5.5±1.2	3.5±0.5	8/4
High/fm qcf down	30.5±2.5/26.8±0.8/3.6±2.7	14.5±2.3/285.1±83.9/0.7±0.1	283.9±256.8/2.8±5.2	5.6±1.6	3.8±1.1	9/4
*Eumops glaucinus*						
Low/fm qcf down	27.4±3.4/19.0±0.4/8.4±3.5	16.2±4.5/321.1±102.7/1.1±0.4	587.8±324.6/5.4±2.0	5.5±2.3	3.7±1.6	37/10
High/fm qcf down	29.3±4.2/20.3±0.3/8.9±4.1	16.7±4.5/270.9±92.8/1.0±0.9	598.8±342.3/6.9±3.7	7.3±4.0	4.3±2.1	16/10
*Eumops auripendulus*						
Low/fm qcf down	32.4±4.3/18.2±1.6/14.3±3.9	20.3±6.9/269.4±68.9/1.5±0.4	777.8±314.2/9.7±3.8	8.4±3.5	4.1±1.2	41/9
High/fm qcf down	35.8±4.1/21.9±1.6/13.8±4.0	19.3±4.0/215.9±61.0/1.5±0.3	769.1±312.9/12.9±8.4	9.5±2.4	5.1±1.6	27/9
*Eumops dabbenei*						
Low/fm qcf down	21.3±1.2/13.7±0.5/7.6±1.1	28.3±2.8/379.9±123.6/1.1±0.3	266.4±30.6/5.4±1.2	9.0±3.2	3.3±1.6	22/6
High/fm qcf down	24.6±2.3/15.8±0.8/8.9±2.9	25.6±1.8/332.7±100.1/1.1±0.2	343.5±93.9/7.0±4.9	8.7±2.7	4.5±1.2	23/6
*Species: F*_17, 295_ =	103.0/29.45/38.1	61.8/64.9/42.9	12.1/34.3	81.8	103.0	
*Genera: F*_7, 295_ =	177.9/41.06/51.9	96.5/110.2/89.1	10.9/60.6	157.6	185.0	

Call type ( =  call frequency alternation) refers to changes in frequency. Call structure ( =  call type alternation) refers to the quasi-constant frequency (qcf) and frequency modulated (fm) components of calls, and to the direction of modulation (down  =  downward; up  =  upward) of calls. SF  =  start frequency; EF  =  end frequency; BW  =  bandwidth; CD  =  call duration; PI  =  pulse interval; D  =  duration fm/qcf; SR  =  sweep rate; S  =  sweep fm/qcf; DC  =  duty cycle; RR  =  repetition rate. Values correspond to the mean ±1 standard deviation, and to the numbers of calls (NC) and sequences (NS) included. *F*-values correspond to sequential tests of parameter contributions to the multivariate distances of species and genera listed in Tables 1, 2, and 3. All *F*-values are highly significant (*p*<0.001).

Finally, we used a Spearman rank order correlation to assess the possible relationship of mean peak frequency, call duration, and pulse interval with forearm length and body mass in all 18 species. These statistical tests were conducted in the “R” statistical package, versions 2.6.1 and 2.8.1 (R development Core Team 2008).

To assess if differences in call frequencies influence prey perception in molossids, we modeled the minimum size of insects to reflect sufficient target strength for detection at several detection distances (1–15 m). Calculations were based on molossid echolocation call peak frequencies, and conducted using the formula: *target strength*  =  *detection threshold* − *source level* − *transmission loss* [40], [41]. We assumed a call intensity of 121 dB SPL [*Molossus molossus*; 42]), and a hearing threshold of 20 db SPL [40], [42]. Environmental conditions were assumed to be 25°C, 80% humidity, and 101325 Pascal. Based on the resulting target strength, we recalculated prey size (TS  = 13.122 log (L) −69,498) [43]. For the interpretation of our results, we only considered prey sizes of 1–40 mm.

## Results

**Figure 2:**
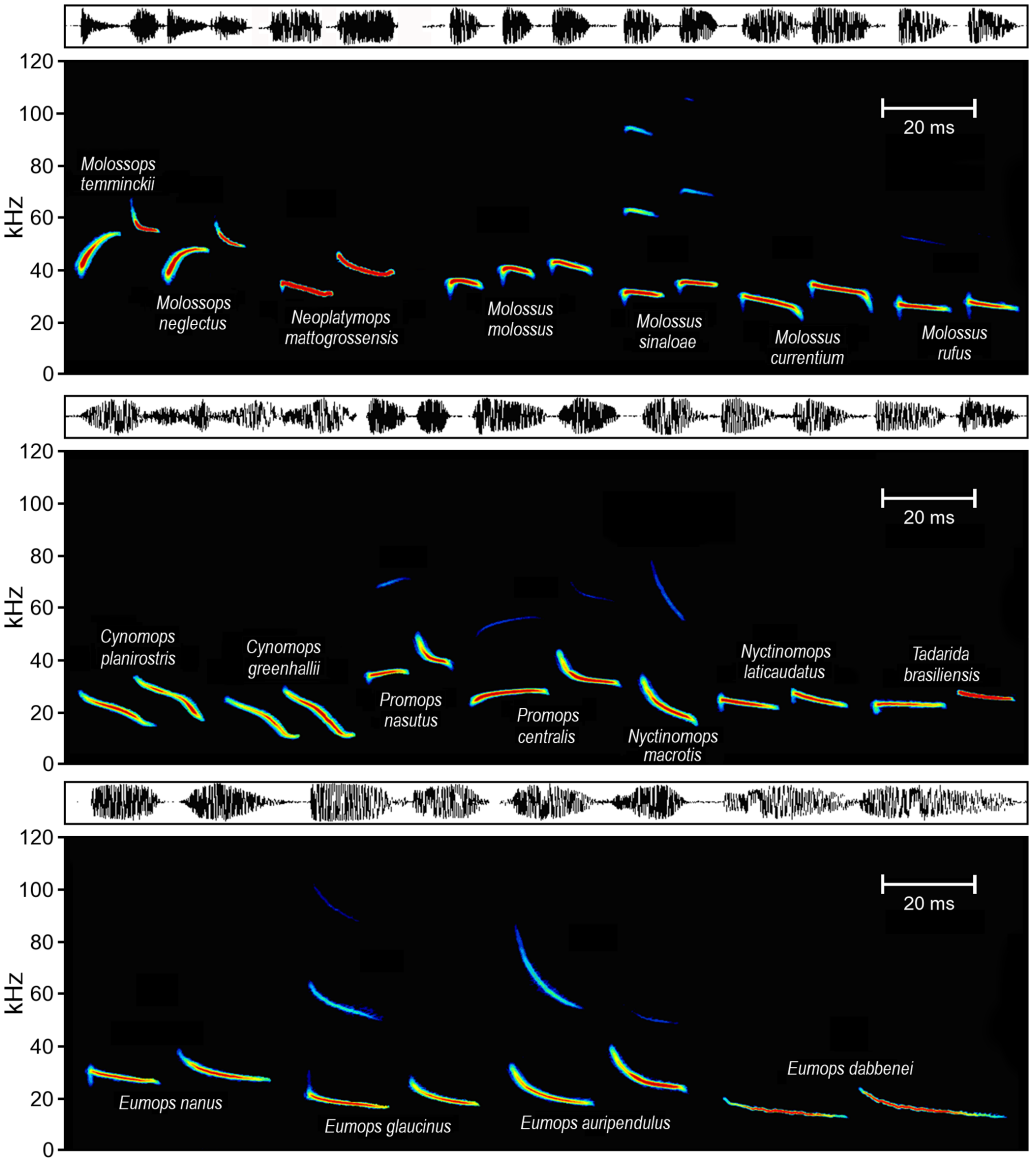
Echolocation call design in Molossids. Spectrograms and oscillograms of typical echolocation calls emitted during search flight by 18 species of New World molossid bats. Call frequency alternation (2 or 3 frequencies) was characteristic for the genera *Eumops*, *Cynomops*, *Neoplatymops*, *Molossus*, and *Nyctinomops*. Alternation between upward and downward modulated call types were observed only in *Molossops* and *Promops*. Species are ordered according to peak frequency and potential flight distance to background clutter. Pulse intervals are not scaled.

Echolocation calls of molossids, with the exception of the genus *Molossops*, show a typical design for open space foraging, with long, shallow-modulated signals emitted at rather low frequencies. Most molossid species revealed high plasticity in echolocation call parameters, emitting echolocation calls with alternating peak frequencies (Table 1, 2, 3). In addition, we observed occasional alternations in call structure (upward/downward modulation) in the genera *Molossops* and *Promop*s (Figure 2, Figure S1).

Despite this high variability and against our expectations, permutational multivariate analysis revealed significant differences in echolocation call design, both among species (*F*_10,295_ = 11.7, p<0.001) and among genera (*F*_7,295_ = 235.9, *p*<0.001). In addition, our analysis revealed that variance in echolocation call design is significantly smaller within than among species (*F*_17,278_ = 6.0, *p*<0.001) and within than among genera (*F*_7,288_ = 9.1, *p*<0.001). These results indicate that New World molossids possess genera-specific and species-specific echolocation call designs.

**Figure 3:**
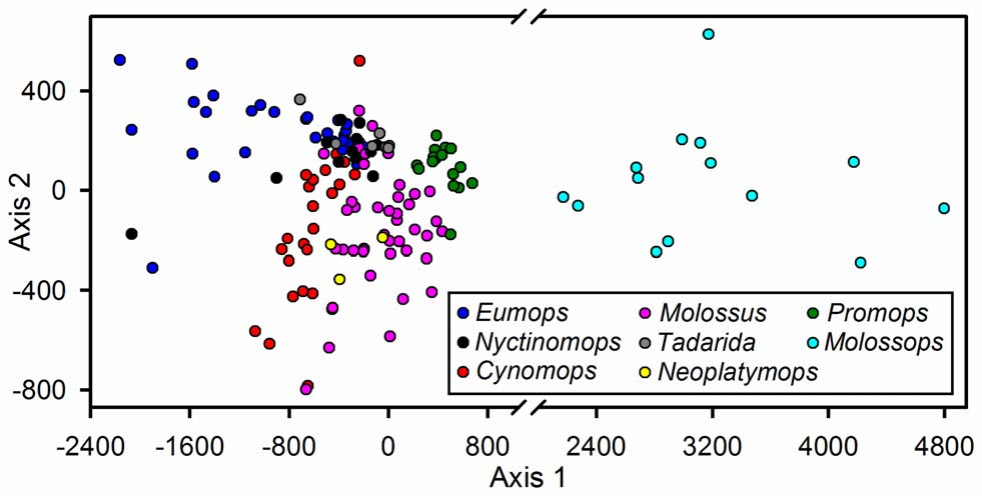
Phylogenetic signature of echolocation calls of Molossids. Principal Coordinate Analysis (PCoA) of 8 genera of New World Molossidae based on Euclidean distances of the structural parameters of their echolocation calls, excluding frequency and sequential time information. Symbols represent sequences (individuals) per species.

Structural echolocation call parameters separated the 18 molossid species in signal space (Figure 3; cumulative sum of the proportion of variance explained, Axis 1 = 0.94, Axis 2 = 0.99). While we mainly observed a transition from downward to upward modulation along Axis 1, slight differences in bandwidth, and in the proportion of the frequency modulated to quasi-constant frequency component, were represented along Axis 2. Despite a considerable overlap among species and genera, echolocation call structure was significantly more similar within than among genera (*F*_7,147_ = 109.7, *p*<0.001).

**Figure 4:**
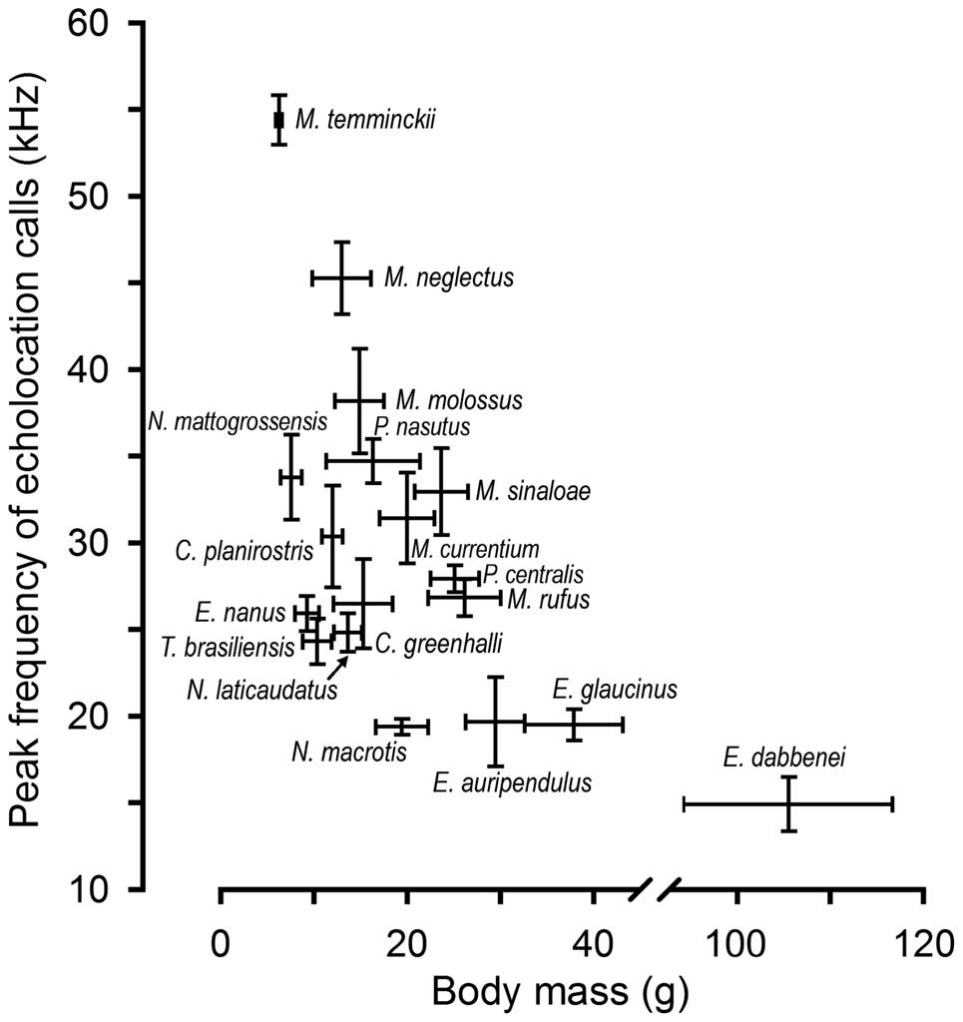
Allometric scaling of echolocation call frequency. Mean and range of peak frequencies (frequencies of maximum amplitude within each signal) versus mean and range of body masses for 18 species of New World molossid bats. For full species names, see Table 1, 2 & 3.

As expected, frequency of search calls in the 18 molossid species is negatively correlated with body size (peak frequency versus forearm length, *ñ* = −0.63, *p*<0.01; peak frequency versus body mass, *ñ* = −0.51, *p*<0.05; Figure 4). With some minor exceptions (*Molossus currentium* vs. *M. sinaloae*; *Eumops auripendulus* vs. *E. glaucinus*), this pattern was consistent within genera, with smaller species calling at higher frequencies than larger congeneric species. In addition, species with higher calling frequencies and smaller forearm length had significantly shorter call duration (peak frequency: *ñ* = −0.74, *p*<0.001; forearm length: *ñ* = −0.67, *p*<0.01), and emitted calls at shorter pulse intervals (peak frequency: *ñ* = −0.83, *p*<0.001; forearm length: *ñ* = −0.58, *p*<0.01). These results confirm allometric scaling of echolocation call parameters in New World molossids, and indicate that tonal and temporal parameters are important contributors to species-specific differences in call design in congeneric species.

**Figure 5:**
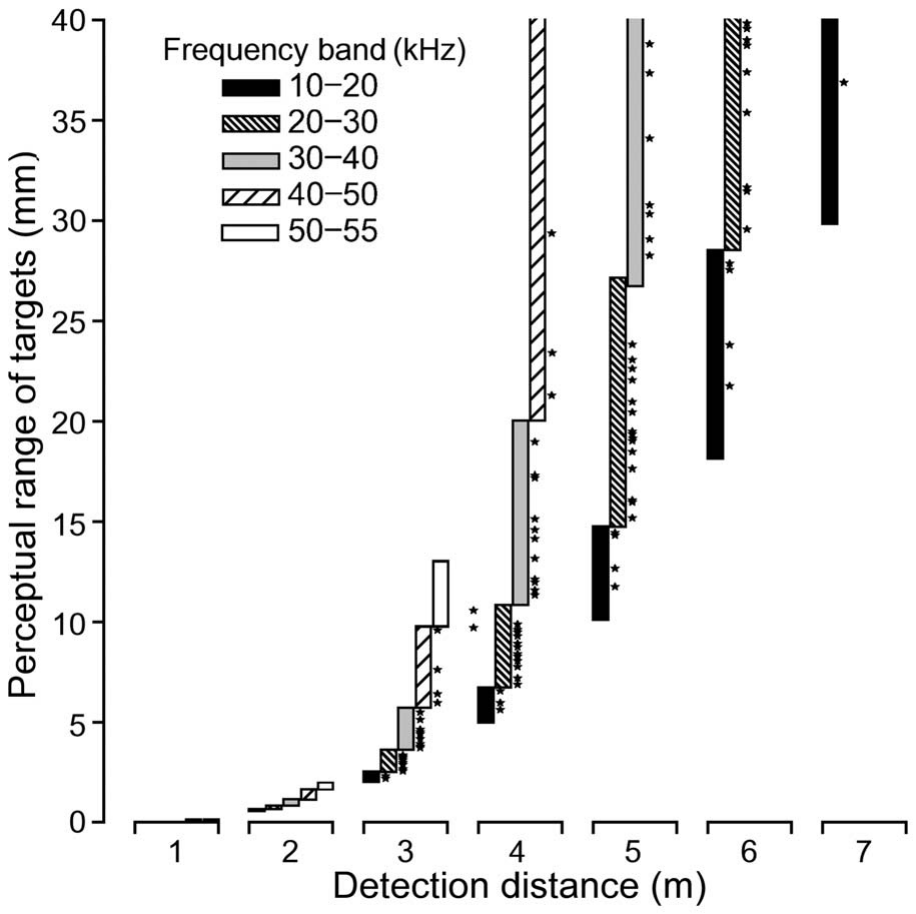
Prey perception. Perceptual range of insects (10–40 mm) with sufficient target strength for detection at different detection distances. Bars represent the perceptual range of targets based on the frequency range between 15 and 55 kHz, separated into five frequency bands (10–20, 20–30, 30–40, 40–50, and 50–55 kHz). Stars represent sizes of detected prey items at different detection distances based on peak frequencies of molossid calls (including frequency alternations; Table 1, 2, 3). Calling intensity was assumed to be 121 dB SPL, and hearing threshold to be 20 dB SPL. In addition, environmental conditions were assumed to be 25°C, 80% humidity, and 101325 Pascal. The model shows that the perceptual range of targets due to frequencies of 20–40 kHz reaches an optimum at 4–5 m.

As expected, echolocation frequency appears to influence prey perception. Our model, which assumes a constant call intensity and detection threshold, shows that higher frequencies lead to a limitation of prey perception towards shorter maximum detection ranges while allowing the detection of smaller (<1.5 cm) prey items. Lower frequencies allow longer detection distances, but restrict prey perception to larger (4 cm) prey items (Figure 5).

In addition, our model reveals that, at shorter detection distances, the frequency range of 15–55 kHz, in which most molossids call (Tables 1, 2, 3), lead to a small perceptual range of targets, with large frequency differences in echolocation calls allowing only little distinction among differently sized targets. In contrast, at longer detection distances, the frequency range of 15–55 kHz leads to a wider perceptual range, with small differences in the frequency domain of echolocation calls allowing a rather good distinction of different prey sizes, implying greater target resolution. Due to atmospheric attenuation effects, the perceptual range for prey items within the size range of 1–40 mm decreased again for larger bat-to-prey distances. This implies that target perception (for prey with body sizes from 1 to 40 mm) due to frequency differences is distance dependent. Considering a constant call intensity of 120 dB and a detection threshold of 0 dB, it reaches an optimum at a bat-to-prey distance of ∼4–5 m (Figure 5).

## Discussion

Understanding how sensory systems have diverged in functional properties at the species level requires a true integration of ecological and evolutionary perspectives [44]. Bat echolocation calls provide remarkable examples of good design through evolution [2]. However, unraveling evolutionary drivers for species-specific signal design in bats has so far been complicated by a limited availability of referenced echolocation calls for related, diverse, and potentially coexisting species sharing similar ecological and sensorial challenges due to habitat and foraging style.

Most molossids (among New World representatives, *Molossops* is an exception) are thought to forage in open space above the forest canopy and savannas, thus facing the main echolocation requirement of long range detection. Therefore, leaving aside foraging style and habitat, which are similar in most species, our data can be used to evaluate how phylogeny, morphology, and prey perception contribute to signal design in members of the family.

As expected, our results reveal that most molossids emit echolocation calls at rather low frequencies, with similar echolocation attributes, such as long call duration, low bandwidth, and long pulse intervals, thus fitting in the typical echolocation call design of open-space foragers [3], [4], [38]. Only *Molossops temminckii* and *M. neglectus* strongly deviated from this general call design with rather short broadband calls emitted at higher frequencies and in shorter pulse intervals. At least *M. temminckii* (the same may be the case of *M. neglectus*) is unusual among molossids because it forages for small insects very close to vegetation edges [31]. Echolocation calls of both species thus rather reflect the need to discriminate prey echoes against cluttered background, while avoiding collision with the vegetation [3], [4].

Within the general open space echolocation call design, our analysis reveals species-specific and genera-specific echolocation call designs. Echolocation call structure was very similar among congeneric species, indicating that, as previously suggested for emballonurids [11], call structure in New World molossids reflects phylogenetic relatedness within the family level. In this context, our results, showing considerable differences in echolocation call structure between *Cynomops* and *Molossops* (Figure 1), are in agreement with molecular data indicating that both genera are divergent lineages meriting separate taxonomic recognition [39], [45]. However, similarity in echolocation call structure does not always indicate phylogenetic relatedness: *Molossops* forms a clade with *Cynomops* and *Neoplatymops*, whereas *Promops* is the sister taxon of *Molossus* [39]; therefore, the upward modulated fm-qcf calls in both *Molossops* and *Promops* most likely represents a remarkable case of convergent evolution.

Our analysis further confirms allometric scaling of tonal and temporal echolocation call parameters at the family and the genus level in New World molossids, which allows species recognition based on echolocation call parameters. Similar results have been found for vespertilionids, emballonurids, and rhinolophids [10], [13], [15], [41], [46]. However, our data also reveal that some molossid species of different genera but with similar body masses have very distinct echolocation call frequencies (Figure 4). Some of these differences in the frequency domain can be explained by a specialization towards specific prey items. Because higher frequencies reduce detection distances while allowing the perception of smaller prey, this might in part explain the high calling frequencies of *M. temminckii*, which is known to feed on small Coleoptera [31]. Conversely, lower frequencies allow longer detection ranges, but only allow the detection of larger prey items, possibly explaining the low calling frequencies of *Nyctinomops macrotis*, which is known to feed primarily on large moths [47], [48].

Despite notable differences in morphology and body mass, 12 of the 18 molossids (*C. greenhalli, C. planirostris, E. nanus, M. currentium, M. molossus, M. rufus, M. sinaloe, N. laticaudatus, N. mattogrossensis, P. centralis, P. nasutus, T. brasiliensis*; Table 1, 2 & 3, Figure 4) emit search phase echolocation calls within a narrow frequency range of 20–35 kHz (Figure 3). This is surprising because the small frequency differences among these species are unlikely to favor resource partitioning. Therefore, we propose that calling in the frequency range of 20–35 kHz allows the detection of a rather ample range of differently sized potential prey items, while maximizing the response time for successful prey capture. This might also explain call frequency alternation in many of the molossid species. We argue that an optimal prey perception window seems a likely reason as our prey perception model reveals that the perceptual range of differently sized targets (for prey of 1–40 mm) is distance-dependent, reaching an optimum at a bat-to-prey distance of ∼4–5 m (Figure 5). In fast flight [49] a detection distance of 4–5 m might indeed be the critical distance at which targets have to be classified as potential prey items to elicit successful capture attempts. It has to be noted that we assumed the same calling intensity for different species, as well as a rather conservative hearing threshold of 20 dB SPL. Call intensity is known to vary among species [16], [42] and flight situations [50], [51], with generally lower call intensities during target approach [50]. The hearing threshold of flying bats is debated [40]. Both factors determine maximum detection distance, and we might have underestimated the echolocation range of molossids during search phase (see Figure S2). Nevertheless, we argue that bat species foraging in similar habitats and possessing similar flight performances adapt their echolocation calls to converge to an optimal prey perception (detection + classification) window. This conclusion corroborates previous studies suggesting that echolocation is a flexible system allowing different bat species to converge to an optimal field of view [16], and to adjust calling intensities to achieve comparable detection distances for prey [42].

Though orientation and prey perception are prominent functions of echolocation, it has been convincingly demonstrated in previous studies that echolocation signals are also used by the bats to convey socially valuable information about individuals, sexes, and species e.g. [17]–[20]. Species recognition is advantageous to evaluate the profitability of hunting grounds and the availability of roosting sites, and it has been shown that such information can be exploited by coexisting ecologically similar species [52]. Although even rather stereotyped echolocation signals of an emballonurid bat allow extracting social information about the sender [19], a high variability in signal design might enhance the communicative potential of echolocation calls. Most molossid species in our study show high plasticity in echolocation parameters. Despite this high plasticity, our data do show species- and genera-specific signatures in echolocation calls, which we explain by phylogenetic relatedness, allometric scaling, and prey perception. However, these factors hardly explain fully the observed plasticity. The high flexibility in echolocation call design of molossids has been noted before many times e.g. [32], [38], [53], and seems to be a typical echolocation trait in this family. Changes in echolocation parameters of molossid, namely in the frequency domain, have been previously related to flight altitude [36], insect noise [35], but also to the presence of conspecifics [22], [54]. Social communication may thus be an additional evolutionary factor shaping echolocation call design in molossids, and the high plasticity might be key to allow social communication among and within species. Considering that molossids are a relatively recent group of extant bats [9], possibly the high variability in echolocation design is an advanced evolutionary trait which allows a flexible adjustment of the echolocation system to meet various sensorial challenges while conserving the sender identity for social communication.

High-flying insectivorous bats, of which typical molossids are the epitome, are rarely caught with conventional inventory methods such as mist-netting, and are difficult to identify to species or even genus level while they forage owing to their similarities in flight style and to their nocturnal habits. As a consequence, high-flying insectivorous bats are underrepresented in most faunal inventories and museum collections, and there are substantial hiatuses in the knowledge of their taxonomy, natural history, and potential as insect pest regulators, particularly for the tropics where they attain high local diversities. Also, as a consequence, there is a very limited knowledge of the species-specific echolocation call characteristics needed for acoustically identifying these bats under field conditions. We thus hope that the availability of detailed information on the echolocation search calls of a representative sample of New World molossids will have a high heuristic value for faunal inventories, for insect pest control applications, and for further studies aiming to understand the diversity and functionality of bat echolocation systems.

## Supporting Information

Figure S1 **Echolocation sequences of molossids during search flight.** Spectrogram of typical search call sequences of 8 molossid genera. Species are ordered according to peak frequency (high–low) and potential flight distance to background clutter (near–far). Pulse intervals are scaled.
(TIF)

Figure S2 **Prey perception considering different hearing thresholds.** Perceptual range of insects (10–40 mm) with sufficient target strength for detection at different detection distances. Bars represent the perceptual range of targets due to the frequency range of 20–35 kHz. Fill patterns represent different hearing thresholds (black  = 20 dB, grey  = 10 dB, white  = 0 dB). Stars represent sizes of detected prey items at different detection distances based on peak frequencies in the frequency range between 20–35 kHz (including frequency alternations; Table 1, 2, 3). Calling intensity was assumed to be 121 dB SPL, and hearing threshold to be 20 dB SPL. In addition, environmental conditions were assumed to be 25°C, 80% humidity, and 101325 Pascal.
(TIF)
