# Supplementary figures and images for: Driving Factors for the Evolution of Species-Specific Echolocation Call Design in New World Free-Tailed Bats (Molossidae)
Source: PLoS One. 2014 Jan 14;9(1):e85279. doi: 10.1371/journal.pone.0085279 (PMC3891751; doi:10.1371/journal.pone.0085279)

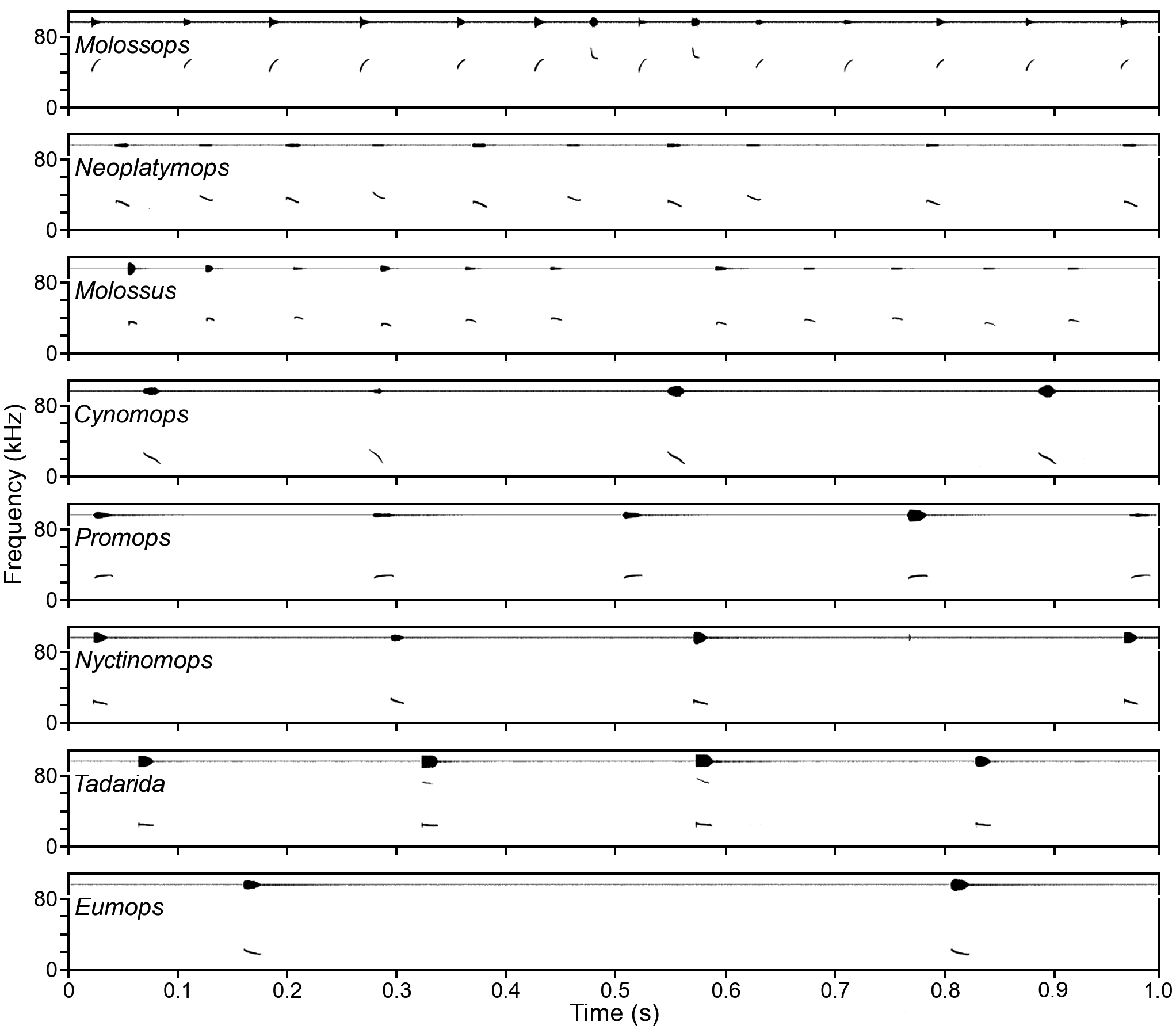

Supplement: Figure S1 — Echolocation sequences of molossids during search flight. Spectrogram of typical search call sequences of 8 molossid genera. Species are ordered according to peak frequency (high–low) and potential flight distance to background clutter (near–far). Pulse intervals are scaled. (TIF) [file pone.0085279.s001.tif]

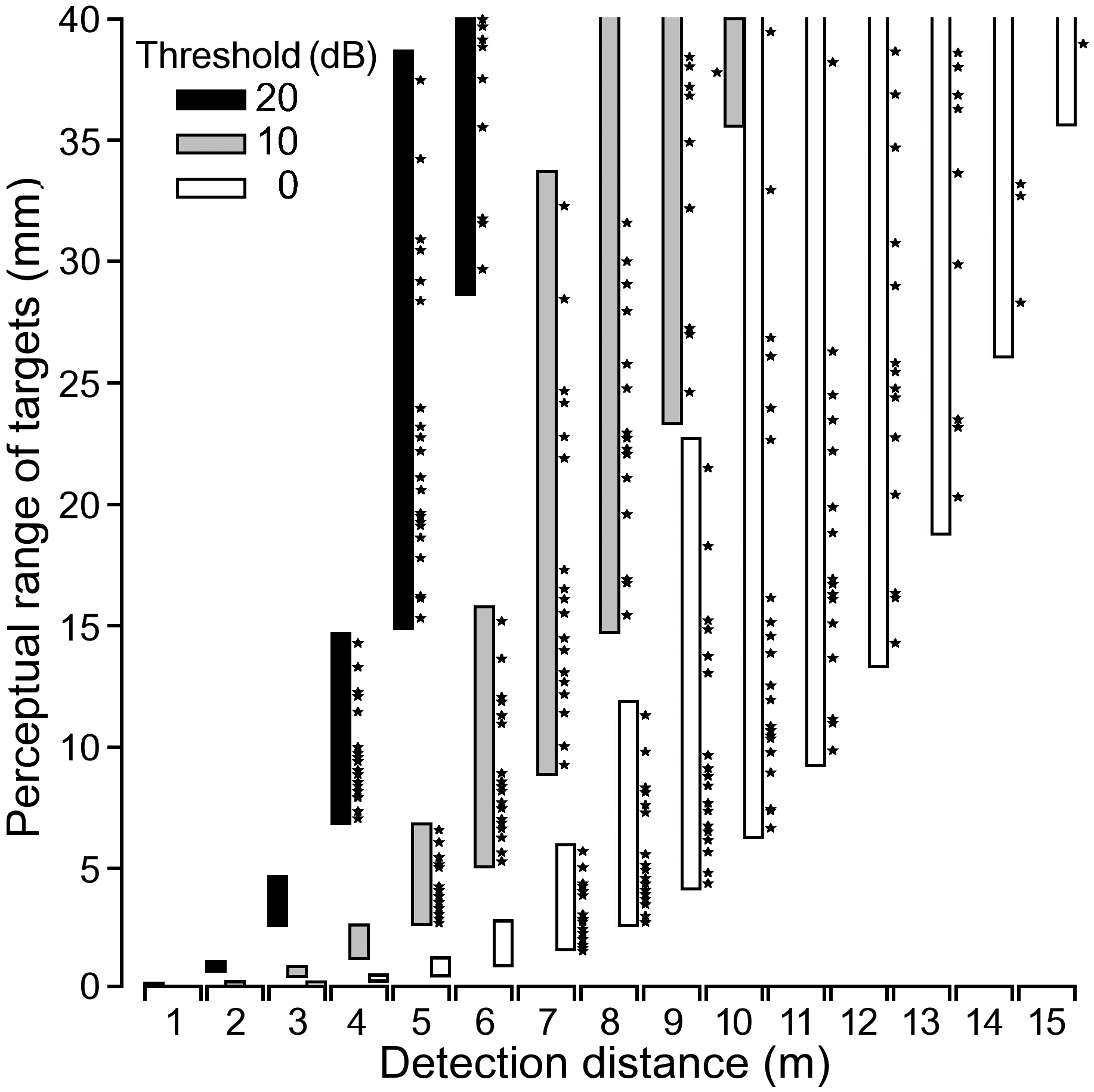

Supplement: Figure S2 — Prey perception considering different hearing thresholds. Perceptual range of insects (10–40 mm) with sufficient target strength for detection at different detection distances. Bars represent the perceptual range of targets due to the frequency range of 20–35 kHz. Fill patterns represent different hearing thresholds (black = 20 dB, grey = 10 dB, white = 0 dB). Stars represent sizes of detected prey items at different detection distances based on peak frequencies in the frequency range between 20–35 kHz (including frequency alternations; Table 1, 2, 3). Calling intensity was assumed to be 121 dB SPL, and hearing threshold to be 20 dB SPL. In addition, environmental conditions were assumed to be 25°C, 80% humidity, and 101325 Pascal. (TIF) [file pone.0085279.s002.tif]
